# A pilot study of circulating microRNAs as potential biomarkers of Fabry disease

**DOI:** 10.18632/oncotarget.25542

**Published:** 2018-06-08

**Authors:** Giuseppe Cammarata, Simone Scalia, Paolo Colomba, Carmela Zizzo, Antonio Pisani, Eleonora Riccio, Michaela Montalbano, Riccardo Alessandro, Antonello Giordano, Giovanni Duro

**Affiliations:** ^1^ Institute of Biomedicine and Molecular Immunology, National Research Council, Palermo, Italy; ^2^ Department of Public Health, Section of Nephrology, Federico II University of Naples, Naples, Italy; ^3^ Department of Biopathology and Medical Biotechnology, University of Palermo, Palermo, Italy; ^4^ Department of Neurology, Guzzardi Hospital, Vittoria, Italy

**Keywords:** Fabry disease, microRNA, biomarker, LVH, ERT, Pathology

## Abstract

Patients suffering from Fabry disease (FD), a lysosomal storage disorder, show a broad range of symptoms and the diagnosis followed by the therapeutic decision remains a great challenge. The biomarkers available today have not proven to be useful for predicting the evolution of the disease and for assessing response to therapy in many patients. Here, we used high-throughput microRNA profiling methodology to identify a specific circulating microRNA profile in FD patients. We discovered a pattern of 10 microRNAs able to identify FD patients when compared to healthy controls. Notably, two of these: the miR199a-5p and the miR-126-3p are able to discriminate FDs from the control subjects with left ventricular hypertrophy, a frequent but non-specific FD symptom. These same microRNAs are also sensitive to enzyme replacement therapy showing variation in the subjects under treatment. Furthermore, two other microRNAs of the profile, the miR-423-5p and the miR-451a, seem useful to highlight cardiac involvement in FD patients. A literature and database search revealed that miR-199a-5p, miR-126-3p, miR-423-5p and miR-451a are known to be linked to pathological states that occur during the FD development. In particular, miR-199a-5p, and miR-126-3p are involved in endothelial dysfunction and miR-423-5p and miR-451a in myocardial remodeling.

In conclusion, in this study we identified a common plasma microRNA profile in FD patients, useful not only for the correct classification of Fabry patients regardless of sex and age, but also to evaluate the response to therapy. Furthermore, our observations suggest that some microRNAs of this profile demonstrate prognostic qualities.

## INTRODUCTION

Fabry disease (FD) is an X-linked, rare, progressive and systemic Lysosomal storage disease. Pathogenic mutations affecting the GLA gene, which encodes the α galactosidase A enzyme, cause the absence or marked reduction of the activity of this lysosomal enzyme, leading to a lysosomal accumulation of globotriaosilceramide (Gb3) and others glycosphingolipids in a wide variety of cell types, especially in microvascular endothelial cells, vascular smooth muscle cells, podocytes and cardiomyocytes [1].

With age, the progressive accumulation leads Fabry patients to renal failure, heart and cerebrovascular diseases and premature death [2]. Fabry patients can manifest two major phenotypes. In men the “classic” manifestations, associated with an absence of enzymatic activity, consist mainly of angiocheratomas, anhidrosis and acropentresiases during puberty, followed by renal failure, left ventricular hypertrophy and cerebrovascular diseases near the fifth decade of life [3]. In men with the “late onset” phenotype, have been identified exon mutations in GLA gene that cause a deficiency of the enzymatic activity but leave a minimal residue of activity. These patients do not have the classic early manifestations but show, in older age, a few isolated symptoms such as left ventricular hypertrophy and renal failure [4]. Women, like men, may develop severe symptoms, but frequently, at an older age and with an unpredictable and insidious disease course, probably due to the random inactivation of the X chromosome [5, 6].

Commonly, artery associated complications (such as cerebral disease and nephropathy) are one of the major clinical manifestations in Fabry disease. Although, the pathophysiology of this specific vasculopathy is unclear, several evidences indicate that the vascular lesions present in Fabry disease occur as a result of endothelial cells dysfunction due to abundant substrate accumulation [7]. Clinical and animal studies demonstrated in FD patients as well as in Fabry mouse model altered endothelial-dependent vascular reactivity [8, 9]. Increased markers of endothelial activation and endothelial microparticles were found in Fabry patients’ plasma [10, 11] as well as enhanced production of ROS [12].

To date, more than 900 mutations have been described in the GLA gene, from which many are private (found in individual family groups). Furthermore, the genotype-phenotype association persists unclear or very weak for many mutations [13]. Even in the same family, the disease can be manifested extremely heterogeneously, and the symptoms may vary from a severe classic to a completely asymptomatic clinical picture [14]. Residual enzymatic activity together with genetic, epigenetic and environmental factors may have a profound influence on the phenotype. Over 15 years of experience with enzyme replacement therapy have shown a correlation of therapy with the slowing in the progression of renal disease, a reduction in the mass of the left ventricle, an improvement in neuropathic pain and, consequently, an improvement in the quality of life [15]. However, the best treatment responses were found in young patients with less organ involvement. That underlines the importance of early diagnosis and treatment to prevent the progression of the disease and the occurrence of irreversible damage [16].

To date, the most efficient diagnostic system for men is the measurement of residual enzyme activity and genetic evaluation. However, in women the value of enzymatic activity is not informative since commonly female Fabry patients show normal values due to the Lyionization of the chromosome X [17]. The accumulation of various substrates, including Gb3 and globotriaosylsphingosine (lysoGb3), have been evaluated as biomarker for FD. In particular, plasma lysoGb3 levels have high diagnostic sensitivity and are likely to correlate with clinical symptoms. However, plasma lysoGb3 does not seem to clearly distinguish asymptomatic and symptomatic individuals among males or females [18, 19]. Therefore, reliable biomarkers for Fabry disease are needed, particularly with regard to female patients.

MicroRNAs are a class of circulating molecules that have been considered as biomarkers only recently. MicroRNAs (miRNAs) are small molecules (22 nt) of non-coding RNA that regulate gene expression at the post-transcriptional level [20]. They bind to target RNA messengers causing their sequestration or degradation [21]. MiRNAs are recognized as important regulators of many physiological and pathological processes. In addition to their intracellular function, recent studies indicate that miRNAs can be exported or released from cells into the bloodstream [22]. Plasma miRNA levels changes in patients with various diseases, especially neoplastic but also metabolic, renal and cardiovascular diseases [23–25]. In this study, we used a “global profiling” approach measuring the levels of 800 microRNAs in a series of Fabry patients, heterogeneous by gender and age group, in order to identify alterations of plasma miRNAs related to the disease.

## RESULTS

### Patient characteristics

We examined a total of 30 Fabry patients (20 treatment naΪve patients with pathogenetic mutations and 10 treated with ERT) and 30 controls (20 healthy subjects and 10 subjects with LVH). Untreated patients and healthy controls were randomly divided into two cohorts. The clinical features of untreated patients, treated patients and control subjects with LVH are reported in Table 1.

**Table 1:** Clinical characteristics of all studied patients: naïve Fabry patients (FD), ERT-treated Fabry patients (ERT), symptomatic controls with left ventricular hypertrophy (LVH) (LVH defined as LVM ≥.110 g/m^2^)

cohort	FD cohort 1	FD cohort2	ERT	LVH
***N***	10	10	10	10
SEX (male/female)	1	1	0, 66	1, 5
age (years)	40 ± 23	32 ± 21	37 ± 9	51 ± 5
**renal manifestations**				
microalbuminuria *N* (%)	20%	30%	10%	0%
macroalbuminuria *N* (%)	10%	0%	0%	0%
**cardiac manifestations**				
LVH *N* (%)	0%	0%	10%	100%
**nervous system involvement**				
stroke *N* (%)	0%	0%	10%	0%
acroparesthesia *N* (%)	30%	60%	30%	0%
**other manifestations**				
angiokeratoma *n* (%)	0%	10%	0%	10%
hypohydrosis *N* (%)	30%	30%	50%	0%

### Fabry patients are characterized by a unique plasma profile

**Figure 1:**
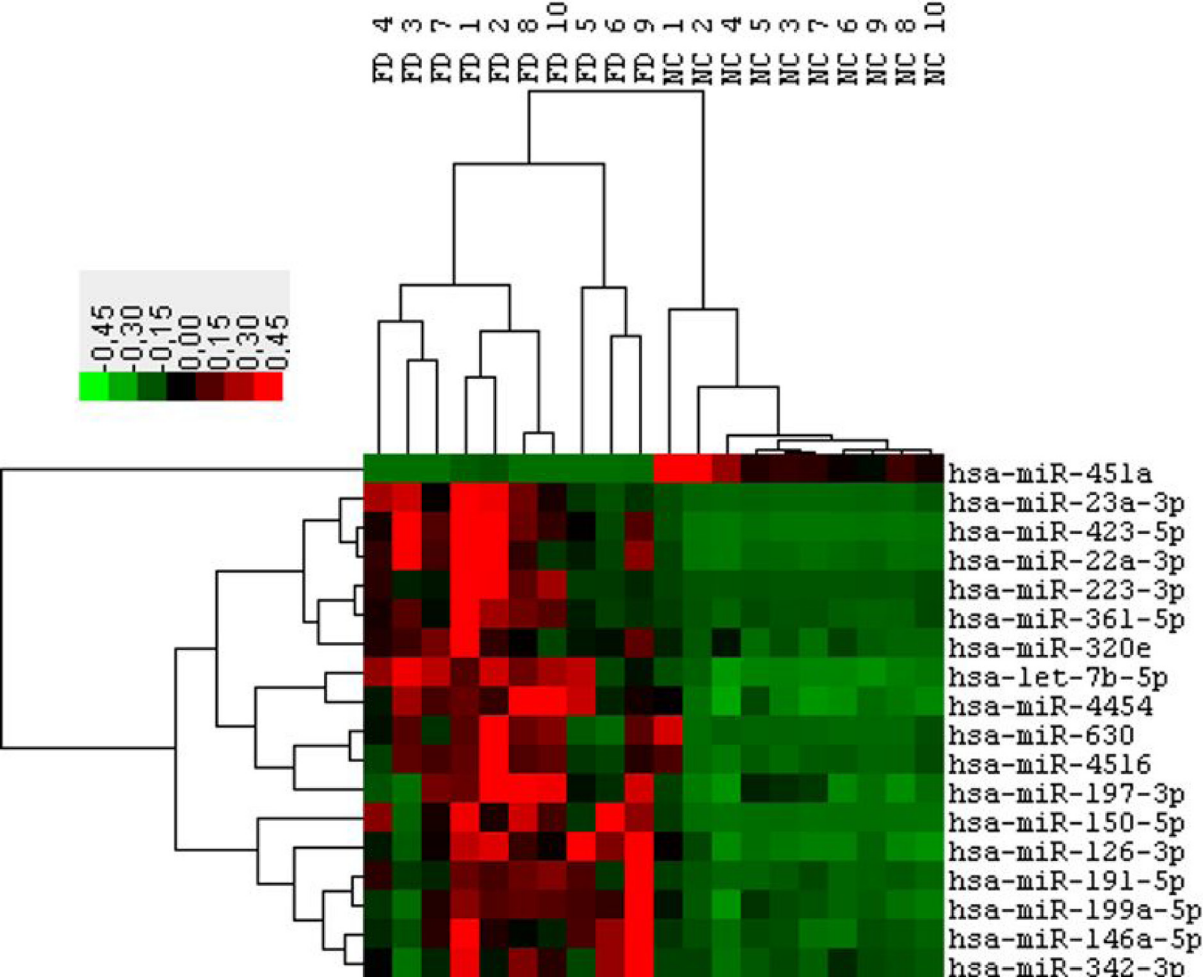
Hierarchical clustering of 18 differentially expressed microRNAs (*P* ≤ 0.05 & FC 1.5) obtained from the Fabry patients (FD) versus normal controls (NC) comparison Data from Nanostring profiling of Cohort1 participants.

The main aim of this study was to generate miRNAs profiles from the plasma fraction of human blood and to determine significant differences in miRNAs between patients diagnosed with Fabry disease (FD) and healthy controls (NC). Total RNA was extracted from 10 FD and 10 NC of the cohort 1. Then, each sample was used for “high-throughput” profiling using the nCounter miRNA expression assay (Nanostring Seattle, USA). The global mean of the most expressed 30 miRNAs was used to normalize the values between the different samples. After the post-normalization and background corrections, the final linear counts were averaged for both the FD and NC samples and the Fold changes were calculated. In this way, we identified 202 miRNAs with mean count levels ≥ 150 (considered an acceptable value superior to the background). Among these 202 miRNAs we identified 18 miRNAs differentially expressed between FD and NC with a fold change of at least 1.5 times and a significance with *p*-value < 0.01 (Figure 1). To validate this result we repeated the measurement of the levels of these 18 miRNAs in the same samples of cohort 1 using a different methodology (RT-qPCR Qiagen). Since there is no unanimous consensus in the choice of control to normalize the values of the circulating microRNAs we focused on miRNAs characterized with the least variation in ranks among the samples as determined by the nCounter assay. We applied an algorithm to determine the top “rank invariant” miRNA and hsa-miR-30a has been identified [26, 27]. This microRNA was subsequently used for the normalization of all RT-qPCR assays. Using the described method, 8 out of the 18 miRNA identified by Nanostring were not confirmed to be connected with FD. The remaining 10 miRNAs showed significant differences between FD and NC with 1 down regulated microRNA (miR-451a) and 9 up-regulated microRNAs (miR-126-3p, 199a-5p, 423-5p, 223, 146a-5p, 23a- 3p, 361-5p, 197-3p, 342-3p) (Table 2).

**Table 2:** Differential expression of validated miRNAs signature for FD and NC samples (cohort 1)

miRNA name	Nanostring cohort 1		RQ-PCR cohort 1	
	Fold change	*P* value	Fold change	*P* value
hsa-miR-451a	0,26	0,0001	0,4	0,0056
hsa-miR-223-3p	37,872	0,0078	8,24	0,0025
hsa-miR-23a-3p	11,504	0,0009	2,986	0,0081
hsa-miR-423-5p	7,875	<0,0001	1,538	0,0087
hsa-miR-361-5p	5,37	0,0058	2,915	0,0009
hsa-miR-126-3p	4,804	0,0001	1,804	0,0044
hsa-miR-146a-5p	4,418	0,0035	3,191	0,0022
hsa-miR-342-3p	3,577	0,0117	2,748	0,0009
hsa-miR-197-3p	2,211	0,001	2,748	0,0064
hsa-miR-199a-5p	2,163	0,003	6,096	0,0001

Comparison of Nanostring and RT-qPCR results.

**Figure 2:**
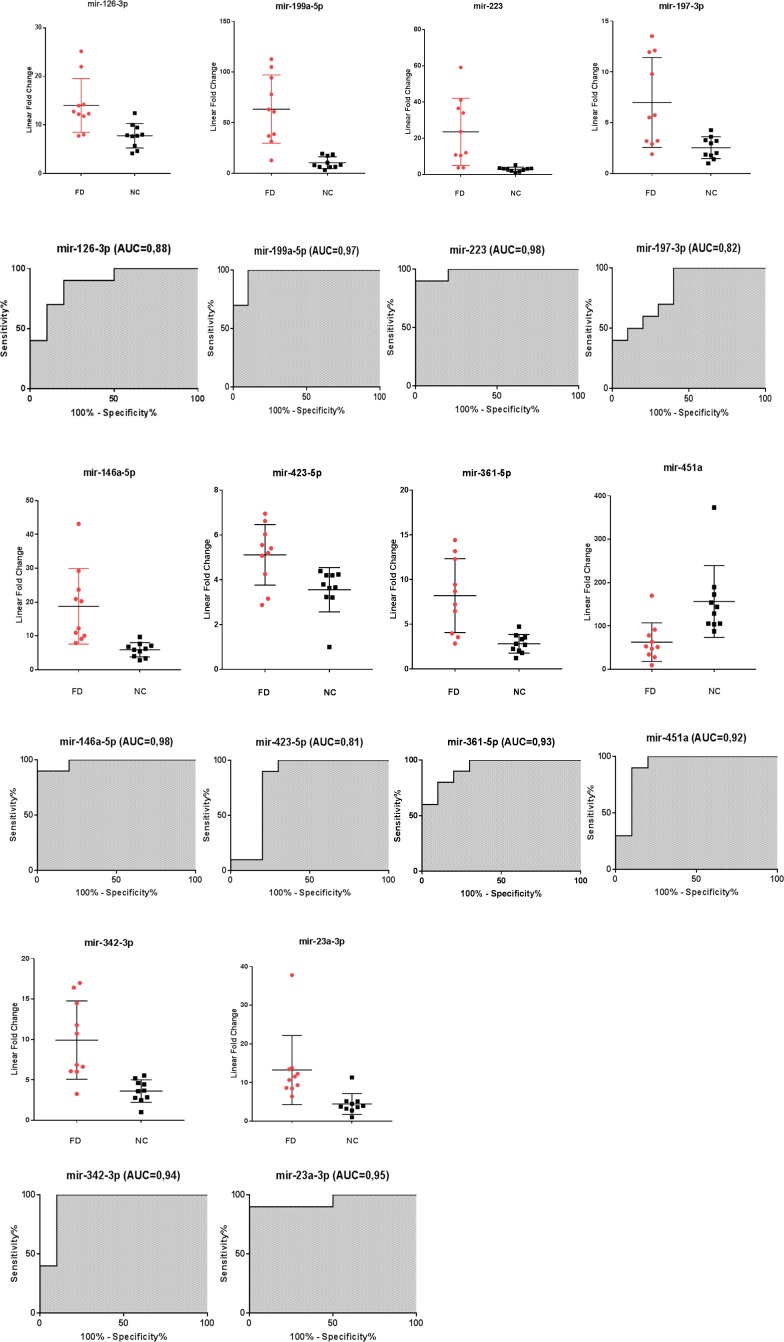
Scatter plots and ROC plots for the 10 validated miRNAs differentiating Fabry patients (FD) and normal controls (NC) (cohort 2) All values were normalized with respect to the lowest samples and plotted on the Y-axis. Data from RT-qPCR assays. (AUC: area under the curve).

When tested in an independent validation cohort (cohort 2) consisting of 10 FD and 10 NC, these 10 microRNAs obtained excellent sensitivity and specificity values, the AUC values are described in (Figure 2).

**Figure 3:**
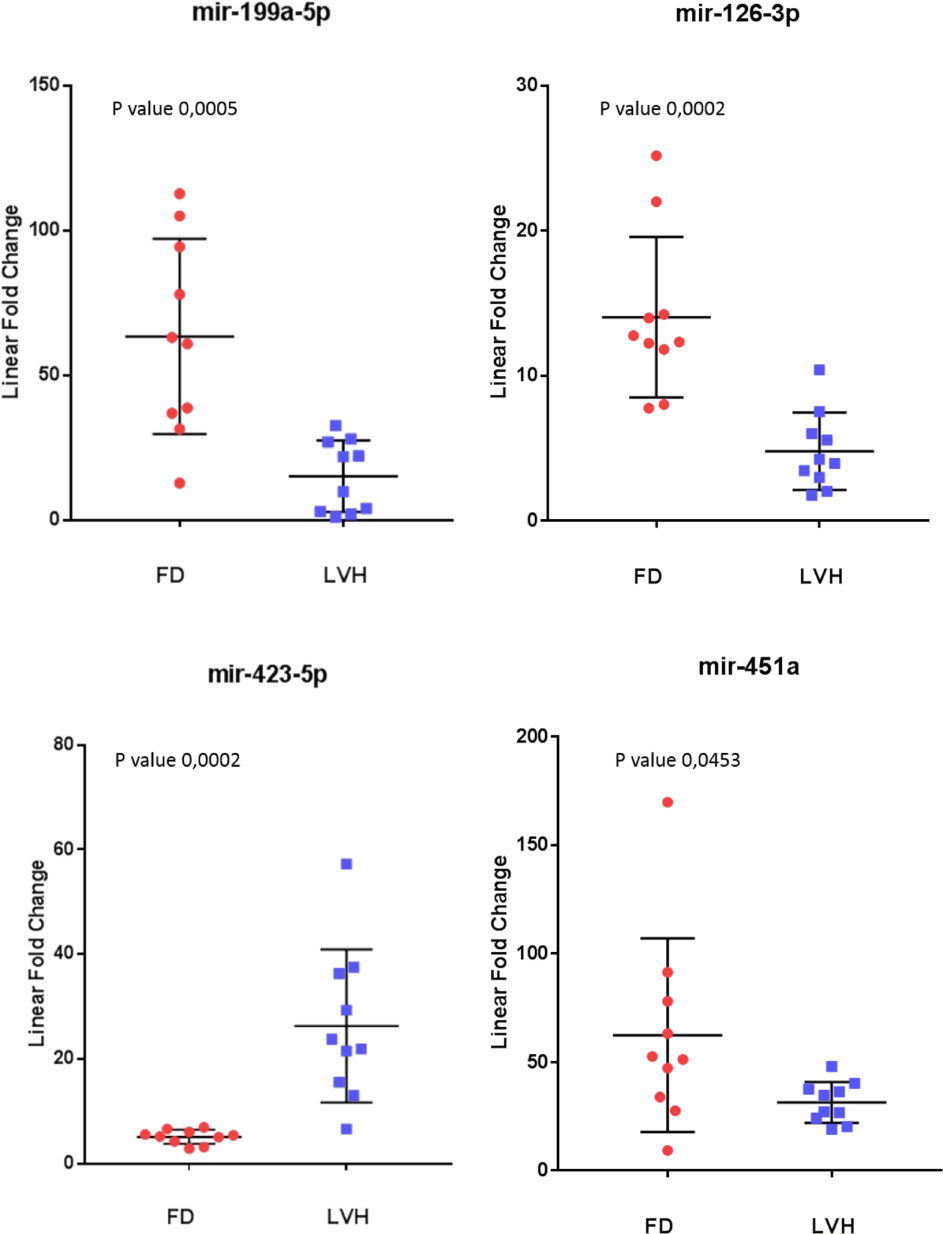
Scatter plots of the 4 miRNAs differentiating Fabry patients (FD) and symptomatic controls with left ventricular hypertrophy (LVH) (*P* value ≤ 0.05) All values were normalized with respect to the lowest samples and plotted on the Y-axis. Data from RT-qPCR assays.

**Figure 4:**
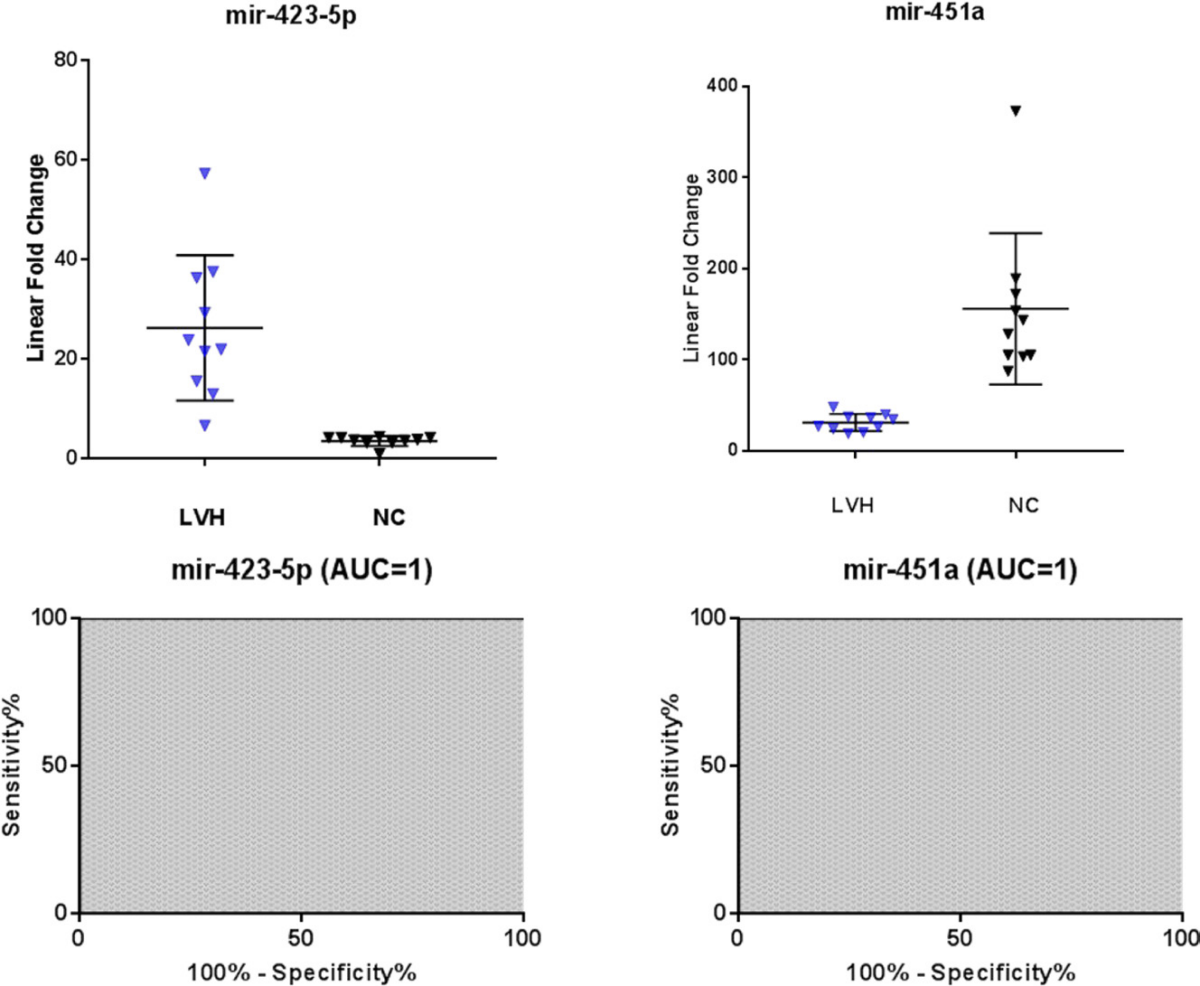
Scatter plots and ROC plots of miR-423-5p and miR-451a levels in symptomatic controls with left ventricular hypertrophy (LVH) with respect to normal controls (NC) All values were normalized with respect to the lowest samples and plotted on the Y-axis. Data from RT-qPCR assays. (AUC: area under the curve).

To further evaluate the specificity of these markers, we compared Fabry disease patients to subjects with similar symptoms but related to other disorders. In particular, we evaluated the levels of the 10 microRNAs in the plasma samples of 10 subjects with left ventricular hypertrophy attributable to causes other than mutations in the GLA gene. Among the 10 previously selected microRNAs we could confirm the differential expression of only 4 microRNAs: 3 up-regulated (miR-126-3p, miR-199a-5p, miR-451a) and 1 down-regulated (miR-423-5p) (Figure 3). It should be noted that the miR-423-5p and miR-451a discriminate subjects with LVH, from healthy controls with high specificity. (Figure 4).

### Effects of enzyme replacement therapy on microRNA levels

To evaluate the effect of enzyme replacement therapy on the 4 microRNAs profile, we analyzed plasma samples of 10 Fabry patients permanently ERT-treated for at least 1 year. It should be noted that, with regard to the clinical characteristics, the treated patients do not differ from those not treated except for a subject under therapy, which shows an evident left ventricular hypertrophy (Table 1).

**Figure 5:**
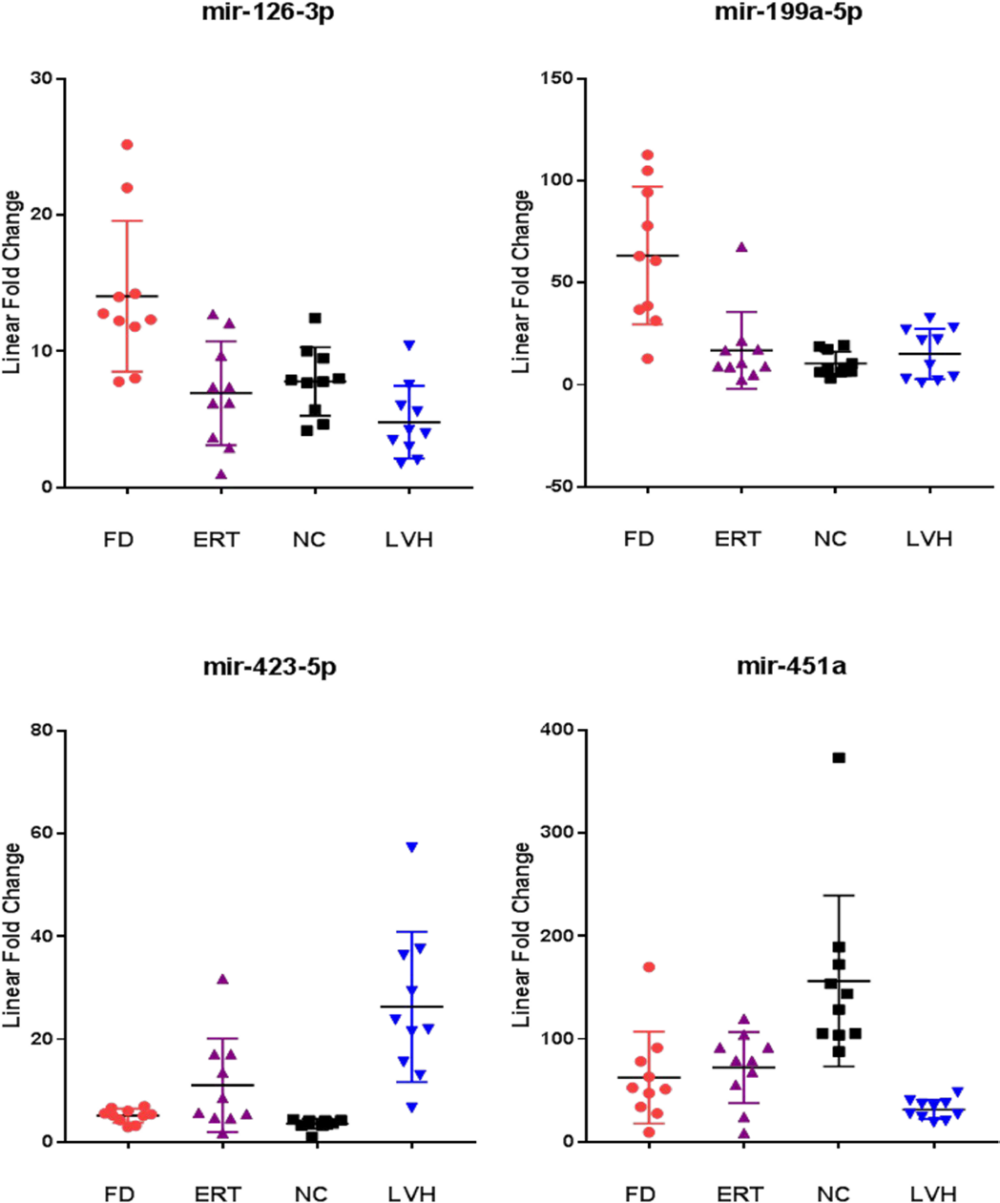
Comparison of the miR-126-3p, miR-199a-5p, miR-423-5p, miR-451a levels from ERT-treated Fabry patients (ERT) with respect to naïve Fabry patients (FD), normal controls (NC) and symptomatic controls with left ventricular hypertrophy (LVH) All values were normalized with respect to the lowest samples and plotted on the Y-axis. Data from RT-qPCR assays.

The results of this analysis described in Figure 5 show that in the subjects under ERT the mean values of miR126-3p and miR-199-5p are lower than those in untreated patients and close to the mean values of the healthy controls. Instead, mean value of miR-451a overlaps that of untreated patients and mean values of miR-423-5 results higher than that of untreated patients.

## DISCUSSION

In this study, we found that Fabry patients, that harbour pathogenetic mutations in GLA gene, are characterized by a specific profile of 10 plasma microRNAs that differentiates them from healthy subjects regardless of the type of mutation, sex and age. Considering the systemic nature of Fabry's disease and the fact that the microRNAs presence in the bloodstream can originate virtually from any cells of all organs and tissues, it is difficult to establish whether the presence of these plasma molecules is linked to the specific pathogenetic mechanism of FD, or instead derived by secondary phenomena common to other disease, such as inflammatory and fibrotic processes [7].

For this reason, we also measured the levels of these 10 microRNAs in the plasma samples of non-Fabry subjects with left ventricular hypertrophy. Left ventricular hypertrophy is a pathological condition often manifested in advanced stages of the disease in Fabry patients of both sexes, although is not a pathognomonic FD symptom and may have many genetic or non-genetic origins [28]. Here, we found that 6 out of 10 microRNAs do not demonstrate significant differences between Fabry patients and subjects with LVH, with mean values overlapping those of FD and significantly higher than healthy controls. This result suggests a link among these microRNAs and a condition pathological but not specific to FD condition. The remaining 4 microRNAs that were significantly differentially expressed between FD and LVH are: miR-126-3p, miR-199a-5p, miR-451a and miR-423a-5p. Notably, miR-126-3p and miR-199a-5p present higher levels in FD than in subjects with LVH with no significant differences between LVH and NC (Figure 5). The last two mentioned microRNAs significantly distinguish Fabry subjects compared to both NC and LVH and suggest a link with specific FD pathophysiological phenomena. Instead, miR-423-5p shows higher levels in LVH compared to FD and higher in FD than in NC (Figure 5) indicating a correlation more specific for the cardiac symptom. Even the miR-451a seems to be linked to the cardiac symptom rather than directly to the Fabry disease. In fact, this miRNA stays more down regulated in the LVH plasma samples compared to the FD and more down regulated in the FD compared to the NC (Figure 5). A systematic literature and database research revealed that miR-126-3p, miR-199a-5p, miR-423-5p and miR-451are involved in pathological processes that occurs also during FD progression [29, 30].

Numerous evidences underline how the miR-126-3p and the miR-199a-5p are both involved in endothelial dysfunction response and in cardiovascular protection mechanisms, resulting deregulated in various diseases in which this phenomenon occurs [31–35]. An increasing body of data has highlighted miR-126, a specific endothelial cells miRNA, as an important regulator of a vascular integrity. Embryonic blood vessel development and angiogenic signaling have been shown to be regulated by miR-126 through the repression of Spred1 [36, 37]. Furthermore, miR-126 counteract atherosclerosis inducing CXCL12 production and influence inflammation by controling endothelial expression of VCAM1 [38, 39]. MiR-199a-5p promotes endothelial cell survival, proliferation and tube formation [40]. In mice cardiomyocytes, miR-199a-5p reduce apoptosis mediating the stabilization of p53. Treatment of mice after myocardial infarction with exogenous miR-199a-5p stimulates cardiac regeneration [41, 42]. MiR-451a and miR-423-5p are both associated with myocardial injury and remodeling process [43], several studies demonstrated altered levels of these miRNAs in the circulation of cardiac patients [44]. MiR-451a is a microRNA mostly expressed in blood and heart [45]. A recent issue showed that miR-451a protects against ischemia/reperfusion-induced cardiomyocyte death by targeting CUG triplet repeat-binding protein2 (CUGBP2)-cyclooxygenase-2 (COX-2) pathway [46]. Importantly, miR-451 regulates autophagy in cardiomyocytes by targeting TSC1 and is decreased in hypertrophic cardiomyopathy showing a negative correlation with left ventricular mass [47]. MiR-423-5p has been widely investigated as a potential biomarker for heart diseases. Several investigations have reported that miR-423-5p is upregulated in human failing myocardium [48]. Another study [49] has demonstrated that circulating levels of miR-423-5p are increased in patients with clinical heart failure, probably, as a consequence from its up-regulation in the myocardium. Although it's controversial, miR-423-5p is considered a biomarker for systemic ventricular and left ventricular remodeling [50]. The association between miR-423-5p and heart disease seems to be linked to the capacity of miR-423-5p to induce apoptosis in cardiomyocytes targeting O-GlcNAc transferase [51]. Summarizing, we have identified, in FD patients, a profile of 4 microRNAs, two of which: the miR-126-3p and the miR-199a-5p are known to be associated with endothelial dysfunction. This phenomenon characterizes the preliminary phases of the FD caused by excessive vascular endothelial accumulation of Gb3 [52]. The other two microRNA: miR-451a and miR-423-5p are probably associated with progressive involvement of the cardiac organ, typical for more advanced stages of the disease [53]. It should be underlined that the levels of miR-451a and miR-423-5p in FD are intermediate between those of the NC and the LVH. This probably because in our series all but one Fabry patients do not have an evident cardiac involvement. At the same time, the intermediate levels between healthy and clearly pathological could suggest an early cardiac involvement in all FD patients not detectable at the clinical level as emerged from recent studies [54, 55]. The involvement of the two pairs of microRNA in two different pathophysiological process could also explain their particular behavior in FD subjects undergoing ERT therapy. With the pair microRNAs related to endothelial dysfunction responding to therapy while those related to organ damage unchanged or increased. In fact, it is known that ERT has a different efficacy on the different symptoms of FD and in particular the regression of symptoms related to the myocardium requires several years of therapy before it can be clinically detected [56].

In conclusion, in this work we have identified a profile of 4 microRNAs that allows accurate identification of Fabry patients regardless of the type of mutation, sex and age and which, at the same time, may be useful for monitoring the response to therapy. Further studies have to be conducted to verify the prognostic potential of these markers and to establish an accurate dose-response relationship between therapy and alterations in these microRNAs levels. Subsequent studies are also needed to understand the role of these miRNAs to distinguish between drivers, those miRNAs which modulate key events conducive to the pathophysiology of Fabry Disease and passengers, those miRNAs whose expression may change as a result of the disease pathogenesis.

## MATERIALS AND METHODS

### Patients characteristics

A total of 60 subjects were recruited: 20 naive Fabry patients, 20 healthy volunteers with normal enzyme activity and no mutations in the GLA gene, 10 subjects with cardiac involvement with normal enzyme activity and no mutations in the GLA gene and 10 permanently ERT-treated patients that were different from the naΪve ones (Table 1). The age and gender of patients were selected to be heterogeneous, while healthy controls were matched by these two parameters. The clinical diagnosis of Fabry disease was confirmed by mutation analysis in all patients. The patients were considered naïve if they harbour a FD causative mutation according to Fabry –database ( http://fabry-database.org/).

The study was carried out in accordance with the Declaration of Helsinki (2000) of the World Medical Association. The Ethical Committee of University of Palermo approved the study protocol and informed consent was obtained from all the subjects. Age, gender, and clinical data of the patients were reviewed and recorded (Table 1).

### GLA gene analysis

Genetic test of the α-GalA gene was performed in all patients as an integral component of their clinical care. DNA samples were extracted from whole blood using a chromatography method (GenElute Blood Genomic DNA Kit, Miniprep, Sigma-Aldrich, USA) and, after the determination of concentrations by spectrophotometer, the amplification of the GLA exons was performed. The PCR products were purified and sequenced using an automated DNA sequencer at BMR Genomics (Padova, Italia).

### α-galactosidase A activity assay

α-galactosidase A activity was measured by Dried Blood Spot test described by Chamoles *et al*. 2001 [57], with minor modifications previously described [58].

### Plasma sample acquisition

Blood samples were drawn using 18-Gauge needle to avoid hemolysis, and they were processed within one hour from the collection to minimize degradation. Plasma was extracted by firstly centrifugation of whole blood at 1900 rpm for 10 minutes at 4°C, and successive centrifugation at 16000 rpm for 10 min at 4°C. All extracted plasma samples were stored −80°C until the miRNA extraction. To minimize the effect of thawing on circulating miRNAs, we only used plasma samples which had not been previously thawed.

### Circulating miRNA extraction

Circulating miRNAs were isolated using the miRNeasy Serum/Plasma kit (Qiagen, Germany) according to the manufacturer's protocol. For every sample, 3 spike-in control: cel-miR-254, osa-miR414, osa-miR442, used for normalization and extraction efficiency control for the Nanostring assay, where added before the extraction. The quality and quantity of the RNA was evaluated by 260/280 ratio using NanoDrop spectrophotometry (NanoDrop ND-1000 Technologies Inc.).

### High-throughput expression profiling of miRNAs

miRNA samples were used as template for the assay “nCounter miRNA expression assay v2” (Nanostring Technology, Seattle, WA-USA) performed in service by Pharmadiagen (Pordenone, Italia). Firstly, the data were normalized for lane-to-lane variation using the provided positive assay controls. Successively, the data were normalized by a global mean normalization using the counts of the 30 miRNA that were more expressed. Each normalized value was then checked to ensure that it was at least 2 SDs higher than the average of background signal recorded for that lane. Any results below this value that was considered zero. Fold change values were then calculated by taking the average of all FD and NC sample expression values for individual miRNAs. Candidate miRNAs were chosen with at least a 1.5 fold difference between average FD and NC samples and average normalized counts 150. We used the expression value of 18 miRNAs (*P* value ≤ 0.05 and at least 1.5-fold expression) to cluster the patients. Hierarchical clustering based on the average linkage of Pearson correlation was employed using Cluster 3.0/TreeView software.

### TaqMan RT-qPCR miRNA assays

The isolated miRNA were retrotranscripted using the “miScript Single Cell qPCR kit” (Qiagen, Germany) according to the manufacturer's protocol. The expression levels of miRNA were evaluated with a SYBR green-based real-time quantitative PCR (RT-qPCR) using the Step one plus (Applied Biosystem, Waltham, MA, USA). For the amplification, we used the “miScript SYBR green PCR kit” (Qiagen, Germany) according the manufacturer's protocol. Triplicate samples and inter-assay controls were used. In this study, we chose as endogenous control miR-30a, the microRNA characterized with the least variation in ranks among the samples as determined by the nCounter assay. Therefore, we used miR-30a for the normalization of our RT-qPCR data using DDCt method. Linear fold changes were then calculated and plotted on scatter plots using Prism (GraphPad PrismSoftware, San Diego, USA).

### ROC curve analysis

The expression profile of each identified miRNA was used as the input for receive operating characteristics (ROC) analysis. ROC curve is displayed as the True Positive Rate (TPR) versus the False Positive Rate (FPR). The area under the ROC exhibit a measure of discrimination accuracy, were calculated using GraphPad PrismSoftware (San Diego, USA).

## Abbreviations

FD: Fabry disease
α-gal A: α-galactosidase A
ERT: enzyme replacement therapy
LVH: left ventricular hypertrophy
ROC: receive operating characteristics curve
AUC: area under the curve
NC: normal control.
